# Case Report: Pallister-Hall syndrome associated with a previously unreported *de novo GLI3* frameshift variant and diagnostic insights from the Middle East

**DOI:** 10.3389/fped.2026.1818628

**Published:** 2026-10-16

**Authors:** Gessila Khalil, Ihab K. Hemieid, Elie G. Malki

**Affiliations:** Faculty of Medicine, Al-Quds University, Abu Deis, Palestine

**Keywords:** case report, genotype-phenotype correlation, *GLI3*, hypothalamic hamartoma, Middle East, Pallister-Hall syndrome, polydactyly

## Abstract

Pallister–Hall syndrome (PHS) is a rare *GLI3*-related disorder characterized by hypothalamic hamartoma and mesoaxial polydactyly. We report an infant from the West Bank, Palestine, with these cardinal findings, imperforate anus, rhizomelic limb shortening, right renal agenesis, choanal atresia, and an ostium secundum atrial septal defect. Whole-exome sequencing identified a previously unreported heterozygous *GLI3* frameshift variant, NM_000168.6:c.2410del (p.Val804SerfsTer5), absent in both parents and therefore presumed *de novo*. The variant lies in the middle-third PHS-associated region and was provisionally classified as likely pathogenic. At the last documented assessment on day 89 of life, no seizures or biochemical evidence of pituitary dysfunction had been identified. Comparison with regional and molecularly relevant reports showed that neurological and endocrine severity varied among patients with truncating variants in this region. This case documents a previously unreported likely pathogenic *GLI3* variant in an established PHS phenotype and supports age-appropriate neurological and endocrine surveillance.

## Introduction

Pallister–Hall syndrome (PHS; OMIM 146510) is a rare autosomal dominant disorder caused by *GLI3* variants and is defined clinically by hypothalamic hamartoma and mesoaxial polydactyly (1). *GLI3* encodes a zinc-finger transcription factor in the Sonic Hedgehog pathway that regulates limb and central nervous system development (2).

Truncating variants in the middle third of the *GLI3* coding sequence (approximately open reading frame nucleotides 1998–3481) are most often associated with PHS, whereas variants producing functional haploinsufficiency more commonly cause Greig cephalopolysyndactyly syndrome (3–7). This relationship is strong but not absolute.

Associated PHS findings include bifid epiglottis, anal atresia, renal anomalies, and other congenital malformations, with variable neurological and endocrine outcomes (1).

We report a Palestinian infant from the West Bank with an established PHS phenotype and a previously unreported, presumed *de novo*, likely pathogenic *GLI3* frameshift variant, and compare the case with regional and molecularly relevant reports.

## Case description

The female infant presented with poor feeding, failure to thrive, and suboptimal postnatal weight gain (Figure 1f). She was delivered by Cesarean section at 38 + 2 weeks after an uncomplicated pregnancy, with Apgar scores of 8 and 9 at 1 and 5 min. Birth weight was 3.02 kg (Z-score = 0), head circumference was 34.5 cm (Z-score = 1.17), and length was 44 cm (Z-score = −2.39). Antenatal screening was unremarkable, and no maternal teratogenic exposure or relevant medical history was reported.

**Figure 1:**
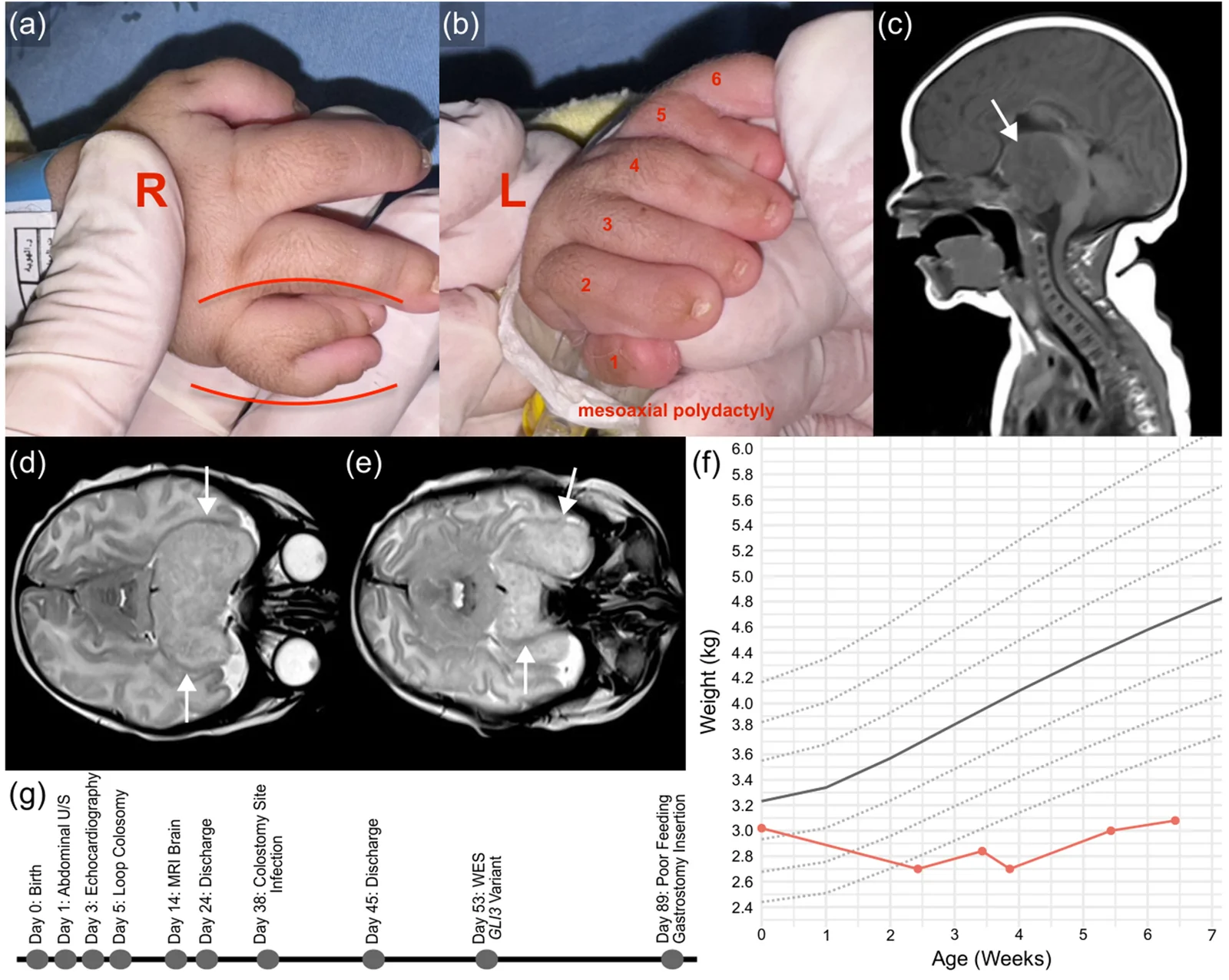
Clinical findings, neuroimaging, growth trajectory, and timeline. **(a)** Right hand demonstrating syndactyly of digits 4–5 (bracket). **(b)** Left hand demonstrating clinically documented mesoaxial polydactyly. **(c–e)** Brain magnetic resonance images showing the hypothalamic hamartoma and associated mass effect. **(f)** Weight trajectory during the first seven weeks of life. **(g)** Timeline from birth through whole-exome sequencing, molecular diagnosis, and gastrostomy insertion on day 89. MRI, magnetic resonance imaging; US, ultrasonography; WES, whole-exome sequencing.

Despite reassuring Apgar scores, the patient developed transient tachypnea of the newborn requiring neonatal intensive care unit admission and supplemental oxygen, which subsequently resolved without complications. Physical examination revealed distinctive craniofacial features, including a depressed nasal bridge, bilateral infraorbital creases, thin vermilion border, microretrognathia, and hirsutism. Skeletal examination demonstrated rhizomelic shortening of the upper and lower limbs, right-hand syndactyly involving the fourth and fifth digits, and left-hand mesoaxial polydactyly (Figures 1a,b). Additional congenital anomalies included a high-arched palate, isolated right choanal atresia (requiring nasopharyngoscopy for diagnosis), and an imperforate anus with a rectovaginal fistula, for which she underwent loop colostomy on day 5 of life.

Imaging studies revealed several significant findings. Echocardiography showed an ostium secundum atrial septal defect. Abdominal ultrasonography demonstrated a solitary left kidney with right renal agenesis; no adrenal tissue was visualized in the right renal fossa. Brain magnetic resonance imaging (MRI) identified a large (5.0 × 3.4 × 3.2 cm), non-enhancing exophytic lesion arising from the tuber cinereum, consistent with a hypothalamic hamartoma. The lesion extended into the prepontine, interpeduncular, and suprasellar cisterns, exerting mass effect on the brainstem with superior displacement of the optic chiasm and compression of the pituitary stalk (Figures 1c–e).

At the time of initial evaluation, the patient had not demonstrated neurological manifestations commonly associated with hypothalamic hamartomas, including gelastic seizures or other paroxysmal events. Baseline endocrine evaluation, including measurement of free thyroxine (FT4), thyroid-stimulating hormone (TSH), growth hormone levels, and gonadotropins, yielded values within age-appropriate reference ranges. Early developmental concerns were noted, including failure to develop a social smile, lack of eye tracking, and inability to bring the hands to the midline; however, tone and primitive reflexes remained intact.

The family history was notable for a 4-year-old brother previously diagnosed with a choroid plexus papilloma and treated with ventriculoperitoneal shunting. Sanger sequencing confirmed that he did not carry the *GLI3* variant identified in the proband. In the absence of a shared variant or an established association between *GLI3*-related PHS and choroid plexus papilloma, the sibling's tumor is considered most likely incidental.

## Diagnostic assessment, therapeutic intervention, follow-up, and outcomes

### Differential diagnosis and clinical reasoning

The combination of anorectal, cardiac, renal, and limb anomalies initially raised consideration of VACTERL association. However, VACTERL does not typically include hypothalamic hamartoma or mesoaxial polydactyly, and the co-occurrence of these two cardinal findings strongly favored PHS. Other *GLI3*-related disorders were also considered. GCPS typically features macrocephaly, hypertelorism or a broad forehead, and preaxial polydactyly; hypothalamic hamartoma is not characteristic. The Greig–acrocallosal spectrum may include preaxial polydactyly, macrocephaly, and callosal abnormalities, whereas the present patient had a large hypothalamic hamartoma and mesoaxial polydactyly without the defining craniofacial pattern of GCPS. Oral-facial-digital and ciliopathy-related disorders can combine polydactyly with central nervous system, renal, or oral anomalies, but the characteristic hypothalamic hamartoma–mesoaxial polydactyly combination and the position of the truncating *GLI3* variant supported PHS. The molecular result therefore confirmed a clinically coherent *GLI3*-related PHS diagnosis rather than VACTERL association or another *GLI3*-associated phenotype (1, 3, 6).

### Genetic findings

Whole-exome sequencing was performed at an external clinical laboratory using the xGen Exome Research Panel v2.0 combined with the xGen Human mtDNA Research Panel v1.0 (Integrated DNA Technologies, Coralville, IA, USA), followed by sequencing on a NovaSeq X instrument (Illumina, San Diego, CA, USA). Secondary analysis was performed using DRAGEN (Illumina) against the hg38 human reference genome, followed by tertiary analysis using the Geneyx Analysis platform (Geneyx Genomex Ltd., Herzliya, Israel). The assay achieved a mean target coverage of 195.8×, with 95.35% of targeted bases covered at ≥20×. Analysis identified heterozygous *GLI3* NM_000168.6:c.2410del (p.Val804SerfsTer5), which was absent from gnomAD v4.1 and ClinVar at the time of analysis (accessed December 23, 2025). Sanger sequencing confirmed the variant in the proband and its absence in both parents and the brother, supporting a presumed *de novo* origin. The frameshift begins at amino acid 804, downstream of the zinc-finger domain, in the middle-third PHS-associated region (Figure 2). RNA and protein studies were not performed. The variant was provisionally classified as likely pathogenic using ACMG/AMP criteria PM1, PM6, PM2_supporting, and PP4; PVS1 was not applied because PHS is associated with a location-specific truncated-repressor mechanism rather than conventional loss of function (5, 8).

**Figure 2:**
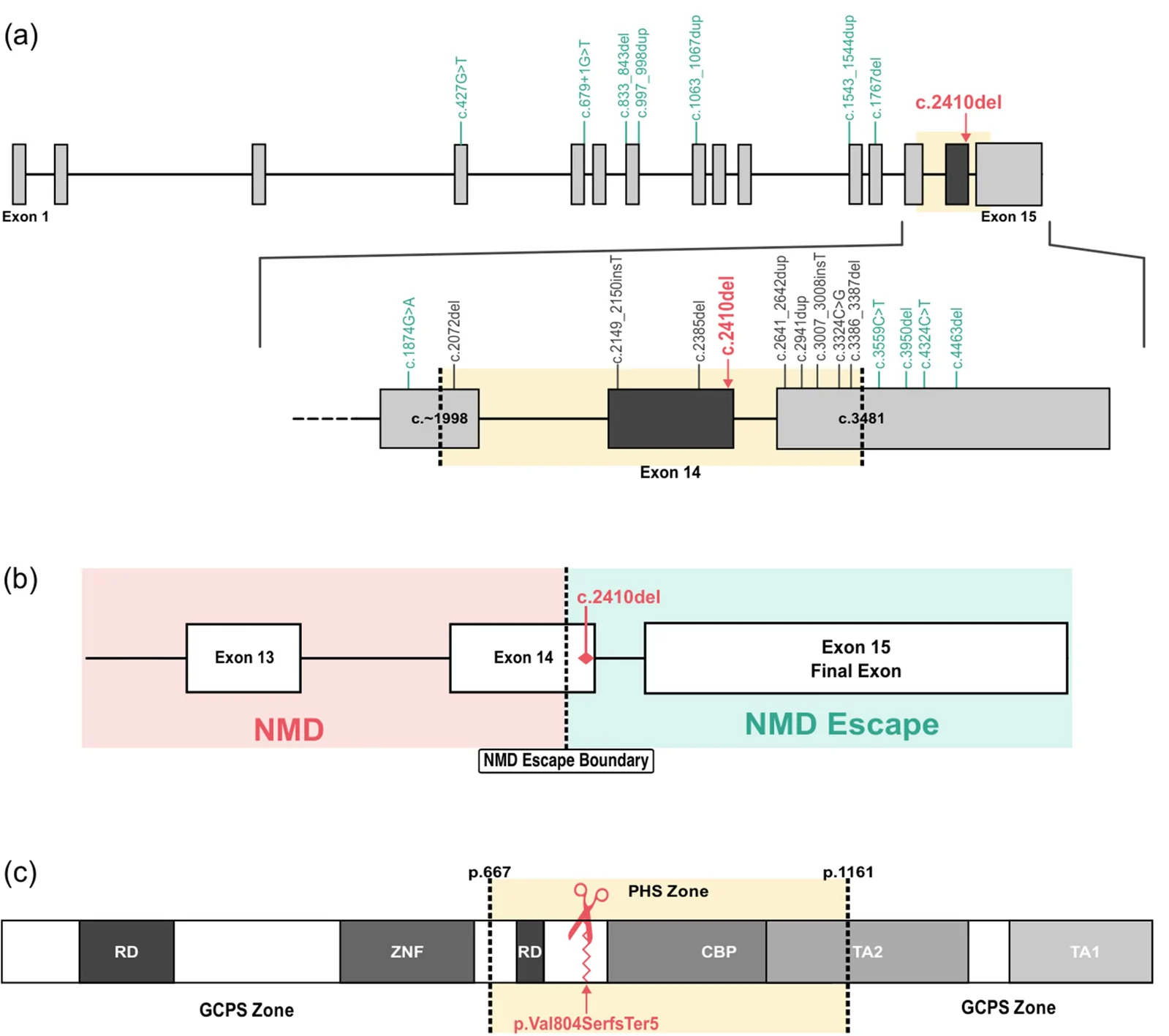
Position and predicted consequence of the *GLI3* variant. **(a)** *GLI3* exon structure and reported variants. **(b)** Location of c.2410del relative to the predicted nonsense-mediated-decay escape boundary. **(c)** Predicted p.Val804SerfsTer5 truncation within the PHS-associated central region. NMD escape and production of a stable truncated protein were predicted but not experimentally confirmed. NMD, nonsense-mediated decay; PHS, Pallister–Hall syndrome; GCPS, Greig cephalopolysyndactyly syndrome.

### Management and clinical course

Management was multidisciplinary. Loop colostomy was performed on day 5 of life for the anorectal malformation, and gastrostomy was inserted on day 89 for persistent feeding difficulty and failure to thrive (Figure 1g). At the last documented assessment, the patient was 89 days old, providing 89 days of follow-up from birth, and was clinically stable without observed seizures. Ongoing surveillance includes neurological and developmental assessment, monitoring of growth and pubertal development, age-appropriate pituitary testing, and neuroimaging as directed by the treating team. Formal laryngeal assessment for bifid epiglottis or laryngotracheal cleft was not documented.

## Comparative analysis of Pallister-Hall syndrome cases from the Middle East

Table 1 compares the present case with regional reports and larger molecular series (5–7, 9–11). The present frameshift begins at p.Val804, close to the Saudi Arabian variant reported as NM_000168.5:c.2396dup, p.(Lys800*) (9). Both patients had hypothalamic hamartoma and polydactyly; however, the Saudi patient developed gelastic seizures and central precocious puberty, whereas neither had been identified in the present patient by the last assessment at 89 days of age (9). The Lebanese clinical case combined hypothalamic hamartoma and polydactyly with orofacial narrowing and tethered cord, although no patient-specific molecular variant was reported (10). In the Iranian family, the homozygous c.1927C > T (p.Arg643Ter) variant was associated with a broader PHS-like phenotype, whereas heterozygous relatives had polydactyly, suggesting an effect of allelic dosage (11). The larger GLI3 series support a strong but non-absolute location-dependent association: c.2385del (p.Ile796fs*13) was reported in PHS near the position affected in the present patient, whereas c.2374C > T (p.Arg792Ter) represents a documented GCPS exception within the PHS-associated region (5–7). Démurger et al. also reported unilateral renal agenesis in a patient with PHS carrying c.2149C > T (p.Gln717Ter), indicating that renal agenesis lies within recognized PHS variability rather than establishing a novel phenotype (7). Differences in age, duration of follow-up, inheritance, and reporting prevent conclusions about variant-specific clinical severity.

**Table 1:** Regional reports and molecularly relevant comparators of Pallister-Hall syndrome and *GLI3*-related disorders. NR, not reported; PHS, Pallister-Hall syndrome; GCPS, Greig cephalopolysyndactyly syndrome.

Report	GLI3 variant and inheritance	Relevant clinical findings	Renal findings
Present case (Palestine)	NM_000168.6:c.2410del (p.Val804SerfsTer5); heterozygous; absent in both parents and therefore presumed *de novo*; provisionally classified as likely pathogenic	Hypothalamic hamartoma, mesoaxial polydactyly, imperforate anus, rhizomelic limb shortening, choanal atresia, and ostium secundum atrial septal defect. No seizures or biochemical evidence of pituitary dysfunction identified by the last assessment at 89 days of age	Right renal agenesis
Omer et al., Saudi Arabia (9)	NM_000168.5:c.2396dup, p.(Lys800*), as reported; heterozygous, likely pathogenic, and confirmed *de novo*	Hypothalamic hamartoma and postaxial polydactyly. Central precocious puberty developed during infancy, gelastic seizures were recognized at 13 months, and central hypothyroidism subsequently developed	No structural renal anomaly reported; renal profile described as normal
Hayek, Lebanon (10)	No patient-specific molecular testing or GLI3 variant was reported; diagnosis was clinical	Hypothalamic hamartoma, postaxial polydactyly, brachydactyly, anal stenosis, choanal atresia, cardiac defect, seizures, growth hormone deficiency, orofacial/esophageal narrowing, and tethered cord	Not reported
Kariminejad et al., Iran (11)	c.1927C > T (p.Arg643Ter); homozygous in the severely affected fetus and heterozygous in relatives with polydactyly	The homozygous fetus had a broad, severe PHS-like phenotype; heterozygous family members primarily had polydactyly	Not reported
Johnston et al. (5, 6)	Large molecular series. Nearby PHS-associated variants included c.2346_2356del11 (p.R782_V786delinsSfsX15), c.2351_2355del5 (p.K784_Q785delinsSfsX15), and c.2483delC (p.P828RfsX14), as originally reported. The recurrent c.2374C > T (p.R792X) variant was associated with GCPS rather than PHS	The 2005 cohort included 135 individuals—89 with GCPS and 46 with PHS. PHS was principally associated with truncating or splice variants in the middle third of GLI3, although clinical and molecular exceptions occurred	Variable or incompletely reported at the individual-variant level
Démurger et al. (7)	Series of 76 individuals from 55 families, including 21 individuals with PHS from 17 families. Relevant variants included c.2385del (p.Ile796fs*13), c.2149C > T (p.Gln717*), and c.2641_2642dup (p.Gln881Hisfs*10), as reported	The two related individuals carrying p.Ile796fs*13 had polydactyly and brachydactyly, but no hypothalamic hamartoma was reported. The p.Gln881Hisfs*10 case had hypothalamic hamartoma, polydactyly, anal atresia, choanal atresia, cardiac anomalies, and developmental findings	Renal anomalies occurred in 41% of evaluable PHS cases (7/17), ranging from renal hypoplasia to agenesis. The patient carrying c.2149C > T (p.Gln717*) had unilateral renal agenesis

## Discussion

The hypothalamic hamartoma and mesoaxial polydactyly establish a clinically coherent PHS phenotype, and the position and predicted consequence of c.2410del support the likely pathogenic classification. Imperforate anus, rhizomelic limb shortening, and the digital abnormalities are established PHS findings. Right renal agenesis has previously been reported within the PHS spectrum, including in the Démurger cohort, and therefore should not be interpreted as a novel or variant-specific association (7, 12). The absence of seizures or endocrine dysfunction at day 89 cannot predict later outcome because these manifestations may emerge with age; longitudinal neurological, endocrine, developmental, renal, and surgical follow-up is therefore warranted.

### Limitations

This single case cannot establish genotype–phenotype causality. Follow-up was limited to 89 days, before some neurological and endocrine manifestations of hypothalamic hamartoma may emerge. RNA or protein studies were not performed, and the comparator reports differed in age, follow-up, and phenotypic detail. Cortisol/ACTH results, formal laryngeal assessment, facial photographs, and palmprints were unavailable.

## Conclusions

This report documents a previously unreported, presumed *de novo*, likely pathogenic *GLI3* frameshift variant, c.2410del (p.Val804SerfsTer5), in a Palestinian infant with an established Pallister–Hall syndrome phenotype. The case adds variant-level evidence and demonstrates the need to interpret molecular position together with phenotype and age at assessment.

### Patient perspective

The patient was unable to provide a personal perspective because of her age. A separate parental perspective was not obtained.

## Data Availability

The datasets presented in this study can be found in online repositories. The names of the repository/repositories and accession number(s) can be found in the article/Supplementary Material.
